# Use of Basalt Fiber-Reinforced Tailings for Improving the Stability of Tailings Dam

**DOI:** 10.3390/ma12081306

**Published:** 2019-04-22

**Authors:** Binbin Zheng, Dongming Zhang, Weisha Liu, Yonghao Yang, Han Yang

**Affiliations:** 1 School of Management Science and Engineering, Shandong Technology and Business University, Yantai 264005, China; rscshbx@163.com; 2 State Key Laboratory of Coal Mine Disaster Dynamics and Control, Chongqing University, Chongqing 400044, China; zhangdm@cqu.edu.cn (D.Z.); yyh369@cqu.edu.cn (Y.Y.); younghame@outlook.com (H.Y.); 3 College of Resource and Environmental Science, Chongqing University, Chongqing 400030, China

**Keywords:** basalt fiber materials, fiber reinforcement, tailings, mechanical property, interface characteristics

## Abstract

As one of the largest artificial geotechnical structures on earth, the tailings dams are classified as one of the high-risk sources in China’s industry. How to improve the stability and safety of tailings dams remains a challenge for mine operators currently. In this paper, an innovative method is presented for improving the stability of tailings dams, in which the basalt fiber is used to reinforce tailings. The mechanical properties of tailings used for dam-construction have a great influence on the stability of tailings dam. In order to investigate the mechanical performance of basalt fiber-reinforced tailings (BFRT), a series of laboratory triaxial tests were conducted. The effects of five parameters (fiber length, fiber content, particle size, dry density and confining pressure) on the mechanical properties of BFRT were studied. The microstructure and the behavior of interfaces between basalt fibers and tailings particles were analyzed by using scanning electron microscopy (SEM). The triaxial experimental test results show that the mechanical properties of BFRT increase with the increases of fiber length and content, particle size, dry density and confining pressure. The SEM results indicate that the interfacial interaction between fibers and tailings particles is mainly affected by particle shape.

## 1. Introduction

Tailings are fine-grained residues left over after the process of separating the valuable fraction from the uneconomic fraction of an ore [1]. Large-scale mining and mineral processing worldwide inevitably produce a significant amount of tailings. Generally, tailings are hydraulically transported into the surface tailings ponds in a slurry form [2]. Tailings ponds are the main way to dispose of solid wastes in China. According to statistics, there are more than 12,000 tailings ponds in China [3]. The storage of tailings has reached 10 billion tons, and this amount is still growing by 0.6 billion tons per year [4]. Tailings dam is an engineered structure based on proper engineering designed for tailings storage. However, many factors, such as slope instability, internal instability, and improper design could contribute to tailings dam failures [5]. These failures have resulted in not only irreversible environmental pollution [6] but also the loss of lives and property in the downstream [7,8]. As can be seen, improving the stability of tailings dams remains a challenge for mine researchers and operators regarding their consequences of failure.

It is generally known that geotextile material is a good geotechnical reinforcement material, playing an important role in improving the performance (strength and stabilization) of soil [9,10,11,12,13,14,15,16,17]. Geofibers have been widely used in geotechnical engineering applications, especially in slopes, embankments and dams [18,19,20]. Park et al. studied the effect of polypropylene fiber reinforcement on the stability of the soil wall [21]. Hong et al. used glass fibers as reinforcing material to reinforce soft soil embankment [22]. Nawel et al. studied the shear strength response on the polyester fibers reinforced silty sand for the slope treatment [23]. Preliminary studies have showed that using geofibers as reinforcing materials had a positive impact on the performance of the geotechnical structures. Basalt fiber, as a new green and environmentally friendly inorganic fiber material [9,24], is mainly applied in concrete, clay soil, and expansive soil reinforcement in geotechnical engineering [25,26,27]. All these studies have shown that the performance of soil can be significantly improved by using basalt fiber as a reinforcing material. The tailings dams are similar to the embankments and slopes in many aspects. Therefore, the application of fiber-reinforced technology in strengthening tailings dam is feasible. At the same time, Chinese technical codes for the design of tailings facilities [28] also encourage the use of safe and environmentally friendly new technologies, such as paste [29,30], dry stacking [31], and some relatively new technologies [4,32,33], to improve the environmental and security stability of tailings dams. These regulations also provide strong support for the application of fibers in tailings disposal. However, no study has been undertaken on the mechanical properties of fiber-reinforced tailings so far, to the best of our knowledge.

Tailings, as artificial sands, are different from natural soils in particle geometry and particle size distribution [34]. The existing studies have shown that the angularity of the tailings particles is much higher than those of the natural sand [35]. This will lead to differences in the effect of fibers on tailings and natural soils. Additionally, tailings are generally discharged into surface tailings pond in the form of slurry [36]. Therefore, the density of fibers is one of the main factors determining whether fiber can be used for tailings reinforcement in order to avoid fiber segregation with the slurry flow. Thus, the existing results cannot be used directly for fiber reinforced tailings, and new experimental research should be carried out. 

This paper presents an innovative method, the basalt fiber-reinforced tailings method, for improving the stability of tailings dams. In order to comprehensively investigate the effect of fiber content, fiber length, dry density, particle size, and confining pressure on the performance of BFRT, a series of triaxial shear tests were carried out. Furthermore, tailings particle shape characterization described as sphericity, convexity, and roughness were obtained quantitatively via image analysis. Based on these particle shape parameters, the interfacial interaction and microstructure between basalt fibers and tailings particles were investigated from the micro-perspective by conducting scanning electron microscopy tests (SEM) to form a preliminary mechanism of the fiber-reinforced tailings.

## 2. Materials 

### 2.1. Characteristics of the Materials

Generally, tailings after ore extraction are transported in a slurry form, which is a mixture of tailings particles and water, into the surface tailings pond. Therefore, the density of the fiber is the key factor in the selection of fibers in order to ensure that the fibers do not segregate in tailings slurry. Although polypropylene fiber is widely used in geotechnical engineering, it is not suitable for tailings reinforcement because its density is less than that of water. Glass fibers have a density similar to the solid tailings, but they are brittle, have poor wear resistance, and are not easy to disperse, so they are also not suitable for tailings reinforcement.

Basalt fiber has a density of 2.7 g·cm^−3^, which is similar to that of tailings particles. It has excellent mechanical properties, corrosion resistance, and acid and alkali resistance [24]. Basalt fiber is a natural and environmentally friendly fiber material, which is a good substitute for other fiber materials in geotechnical engineering. Basalt fiber was selected to reinforce the tailings, and the mechanical properties of BFRT were investigated.

**Figure 1:**
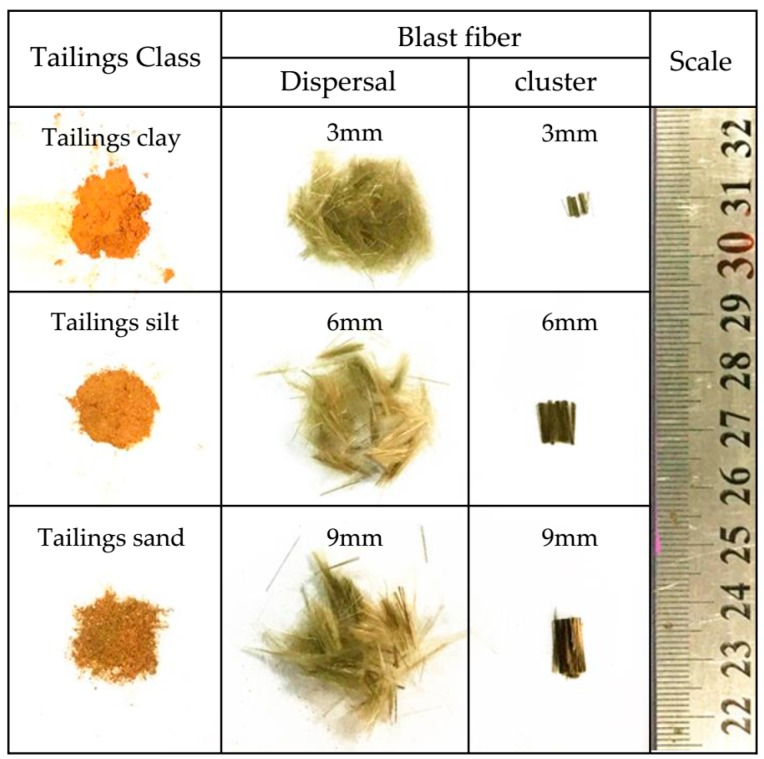
Basalt fibers and tailings particles.

In the experiment, the parameters of monofilament are 17 μm in diameter and 3 mm, 6 mm and 9 mm in length (Figure 1). Table 1 shows the technical specifications of the basalt fibers.

**Figure 2:**
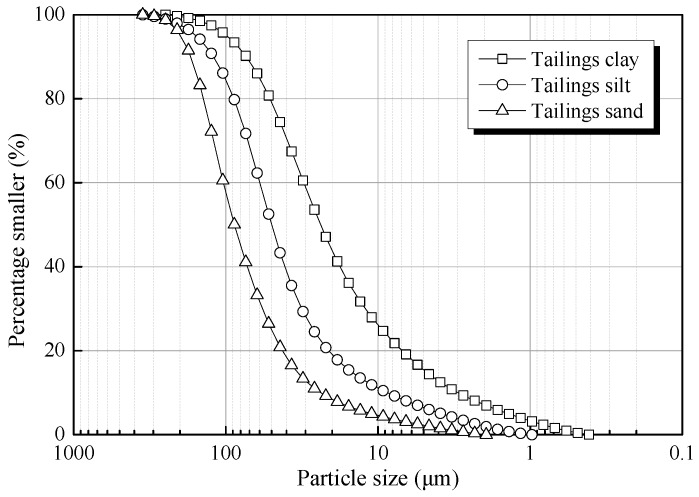
Particle size distribution of the tailings samples.

The test tailings were sampled from Kafang Tin mine (Gejiu, China). The original tailings were classified into three classes using cyclone classification technology. The particle size of each class of the tailings was determined by an S3500 light-scattering particle-size analyzer (made by Microtrac, Inc., Montgomeryville, PA, USA). The particle size distribution of the tailings samples is listed in Figure 2, and the main physical properties are summarized in Table 2. Further, Table 3 presents the main chemical compositions of test tailings obtained through XRF analysis. 

According to the national Technical Code for Geotechnical Engineering of Tailings Embankment (GB50547-2010), the tailings are divided into three classes and seven sub-classes based on their size distribution. They are tailings clay (the sub-classes are silty clay and clays), tailings silt (including silts), and tailings sand (the sub-classes are gravelly sand, coarse sand, medium sand, fine sand, and silty sand) [34]. Based on the above experimental results, the three classes of test tailings can be named as tailings clay (#1), tailings silt (#2), and tailings sand (#3), respectively.

### 2.2. Particle Shape Features

**Figure 3:**
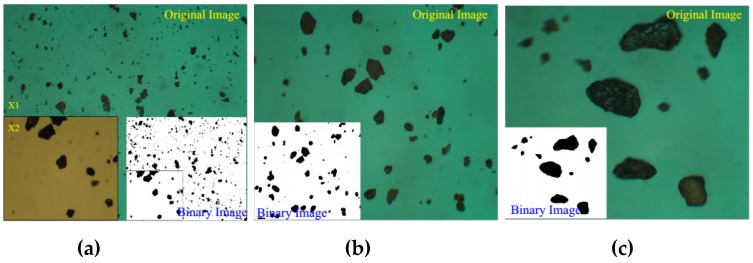
Tailings particle shape from the microscopic perspective. (**a**) silt clay; (**b**) silt sand (**c**) fine sand.

**Figure 4:**
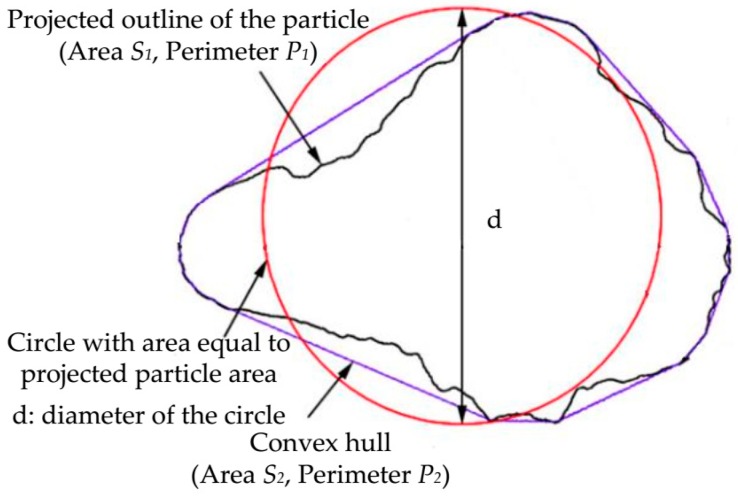
Basic measurements of a particle [35].

In order to investigate the mechanical properties of BFRT from the microcosmic angle, the particle shapes of the test tailings were analyzed by XPV-909E polarizing microscope (Shanghai Changfang Optical Instrument Co. LTD., Shanghai, China) and ImageJ software. The ImageJ software is a Java-based image processing software, and the function of software could be extended through plugins. Figure 3 shows the three classes of tailings particle images, with the same magnification. Quantitative index analysis was conducted to obtain the curve of the particle shape parameter. Particle shape features could be described from three aspects, namely, sphericity, convexity, and roughness. Sphericity is used to quantify the similarity between a particle and a sphere. Convexity is closely related to the angularity of a particle. Roughness is used to describe the fluctuation of the projected outline of a particle. They can be determined by Equations (1)–(3), respectively [35]. And Figure 4 shows the schematic diagrams of those basic measurements.

(1) $$\mathrm{Sphericity}\;=\;\sqrt{4\pi S_{1}}\;/P_{1}$$

(2) $$\mathrm{Convexity}\;=\;S_{1}/S_{2}$$

(3) $$\mathrm{Roughness}\;=\;{\left(P_{1}\;/\;P_{1}\right)}^{2}$$

where, $S_{1}$: Area of the particle outline (mm^2^);

$S_{2}$: Area of the convex hull (mm^2^);
$P_{1}$: Perimeter of the particle outline (mm);
$P_{2}$: Perimeter of the convex hull (mm).

**Figure 5:**
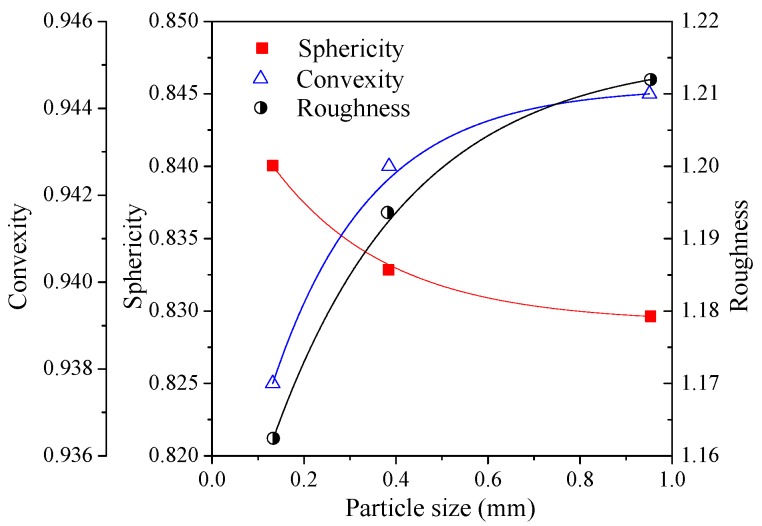
Curve of tailings particle shape parameters.

Figure 5 presents the quantitative index curve of the tailings particle shape parameter. The figure shows:

1. The sphericity of the tailings particles increases with the decrease of particle size, which indicates that the tailings particles tend to be spherical from coarse to fine;
2. The convexity and roughness of the particles increase with the increase in particle size, which indicates that the larger the particle is, the rougher its surface is.

## 3. Testing

### 3.1. Specimen Preparation

The test specimens are cylinder-shaped with a diameter of 39.1 mm and a length of 80 mm. The preparation obeys the following procedures. First, the tailings samples were mixed to predetermined moisture contents and then sealed for 24 h for further mixing. Second, the basalt fibers were uniformly dispersed into the mixed tailings in a predetermined content. Then, the required quantity of the mixtures was placed inside the cylindrical mold for specimen casting using a layer-by-layer compaction method. To ensure uniformity, the compaction process was compressed in four steps. The test samples preparation is completed, and then the triaxial test is carried out.

### 3.2. Test Schemes

The purpose of a triaxial compression test is to investigate the mechanical properties of BFRT. Five key influential factors (fiber length, fiber content, particle size, dry density and confining pressure) that affect the strength behavior of BFRT were explored. Table 4 presents the test schemes. The tests were divided into three groups. Group 1 was used to investigate the effect of fiber parameters (fiber length and fiber content) on the strength behavior of BFRT. The different values for fiber length are 3 mm, 6 mm, 9 mm and fiber contents (FC) are 0.2%, 0.4%, 0.6% by weight of tailings (FC = W_fiber_/W_tailings_). Group 2 was used to investigate the impact of dry density. The dry density of tailings was determined by a consolidation test. The dry densities of tailings clay are 1.40, 1.52, 1.61 g·cm^−3^ and tailings silt are 1.52, 1.60, 1.67 g·cm^−3^ and tailings sand are 1.52, 1.59, 1.65 g·cm^−3^. Group 3 was used to investigate the impact of confining pressure. The confining pressures in this study are 200, 400, 600 kPa. The representative test samples are selected from the failure specimens for SEM analysis.

### 3.3. Test Procedures

According to the above test schemes, a series of triaxial compression tests were conducted under the condition of an undrained consolidation using a TSZ-6A automatic tri-axial apparatus (made by Nanjing soil instrument Co. Ltd. Nanjing, China). The triaxial tests obey the following procedures. (1) Mounting specimen: the test specimen is mounted in the triaxial chamber. (2) Saturation: after assembling the triaxial chamber, sample saturation is performed by applying back pressure to the specimen pore water. (3) Consolidation: this step makes the specimen to reach equilibrium in a drained state at the effective consolidation stress. (4) Shear: after stabilization by consolidation, the axial load is applied to the specimen using a rate of axial strain of 0.4 mm/min. Specimen drainage is not permitted during shear. The whole shear process was automatically controlled by computer to realize a real-time acquisition of test data. The failure of specimens is often taken to correspond to the maximum principal stress difference (maximum deviator stress) attained at 15% axial strain in accordance with the Specification for Soil Test [38]. The maximum deviator stress corresponding to this point was defined as peak strength in this paper. The SEM analyses were performed on TESCAN Mira3 LMH field emission scanning electron microscopes, with the optical system, vacuum system, and imaging system. The resolution ratio can reach 1 nm.

## 4. Experimental Results and Analysis

### 4.1. Effect of Fiber Parameters on Strength Behavior of Tailings

**Figure 6:**
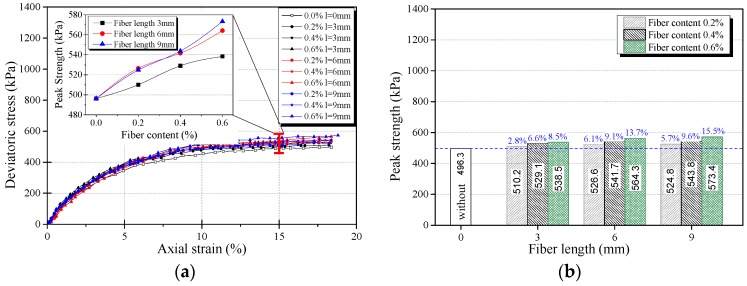
Stress strain curve and peak strength of fiber-reinforced tailings clay (*ρ_d_* = 1.52 g·cm^−3^). (**a**) Stress-strain curve, (**b**) peak strength increment of BFRT.

**Figure 7:**
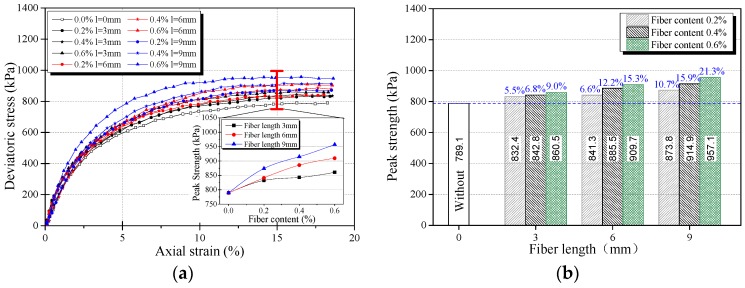
Stress strain curve and peak strength of fiber-reinforced tailings silt (*ρ_d_* = 1.60 g·cm^−3^). (**a**) Stress-strain curve, (**b**) peak strength increment of BFRT.

**Figure 8:**
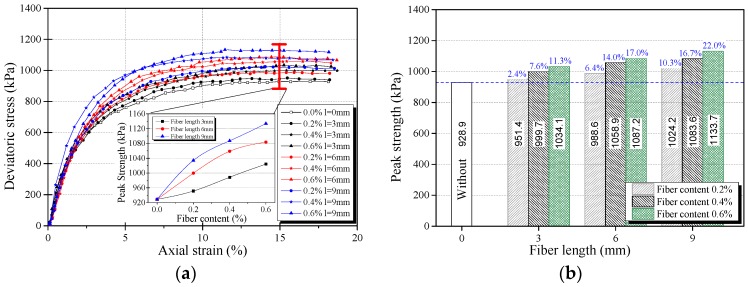
Stress strain curve and peak strength of fiber-reinforced tailings sand (*ρ_d_* = 1.59 g·cm^−3^). (**a**) Stress-strain curve, (**b**) peak strength increment of BFRT.

The stress-strain curves obtained from triaxial compression tests are given in Figure 6a, Figure 7a and Figure 8a for reinforced and unreinforced tailings under 400 kPa confining pressure. Figure 6b, Figure 7b and Figure 8b show the incremental percentage of peak strength of the reinforced tailings compared with unreinforced tailing.

Fiber length and fiber content have a great influence on the mechanical properties of BFRT. Figure 6 shows that the peak strength of the reinforced tailings clay is improved to a certain extent compared with the unreinforced tailings clay. The incremental percentages of peak strength range from 2.8–15.5% with the increase in fiber length and content of the tailings clay. It can be seen from Figure 7 that the peak strength of tailings silt significantly increases with the content and length of fibers, by between 5.5% and 21.3%, and the maximum increment occurs when the length of fibers is 9 mm and the content is 0.6 wt%. Figure 8 shows that the peak strength of fiber-reinforced tailings sand also increases significantly compared with the unreinforced tailings sand. The peak strength of unreinforced tailings sand is 928.9 kPa, and it reaches to 1133.7 kPa after adding 9 mm fiber with the content of 0.6 wt%. The increment of the peak strength is 204.8 kPa, the corresponding incremental percentage is 22%.

Figure 6, Figure 7 and Figure 8 shows that the fiber reinforcement tended to increase the peak strength of the specimens. In addition, the peak strength improved gradually with the increase in fiber content and fiber length. Ranjan et al. [39], Heineck et al. [40], and Casagrande et al. [41] also reported that the inclusion of the fibers can significantly improve the strength of the soil and improve the ductility of soil. These results are consistent with the findings of BFRT. The following results are concluded:

1. Under the same fiber length, the peak strength of BFRT improves with the increase in fiber content. The reason for this is that with the increase in the fiber content, the fibers in tailings gradually form a spatial network system from a scattered distribution.
2. When the fiber content is the same, the peak strength of the BFRT improves with the increase in the fiber length. The reason for this is that the increase in fiber length makes it easier for the monofilaments to lap into nets.

**Figure 9:**
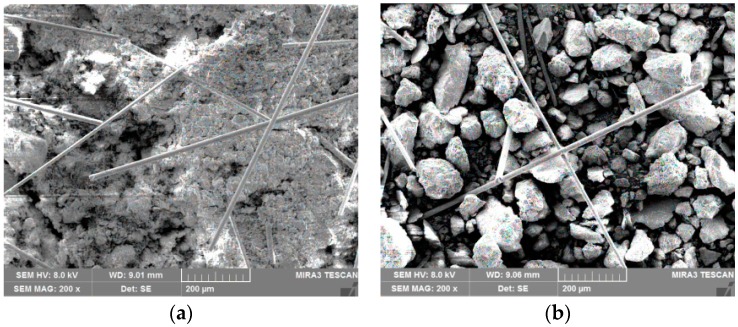
Fiber-network structure in BFRT: (**a**) tailings clay with 0.6% fiber content, (**b**) tailings sand with 0.4% fiber content.

The network structures in tailings can effectively bear the pulling force and prevent the destruction of the tailings specimens. Figure 9a,b are network structure formed in tailings clay and tailings sand with a fiber length of 6 mm and fiber content of 0.6 wt% and 0.4 wt%, respectively. It can be seen that the increase in fiber content tends to form more network structures.

### 4.2. Effect of Dry Density on Strength Behavior of Tailings

**Figure 10:**
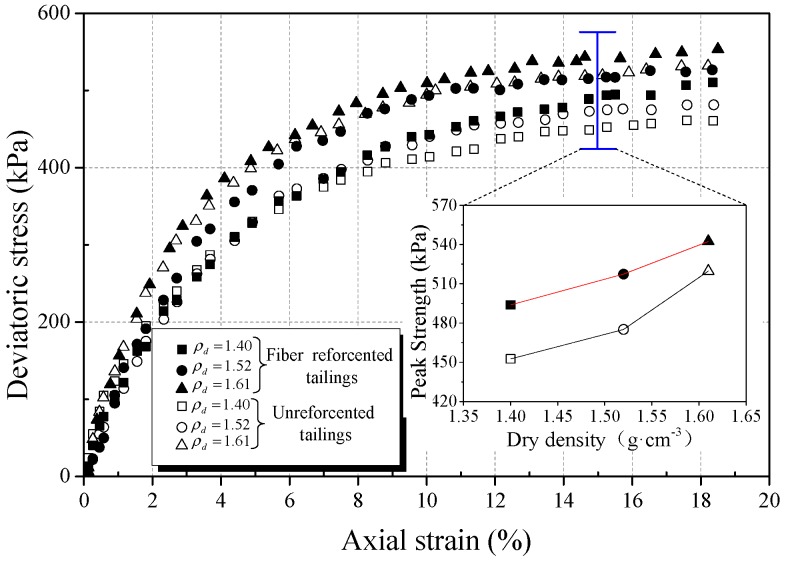
Effect of dry density on mechanical behavior of reinforced tailings clay (0.4 wt% fiber content).

**Figure 11:**
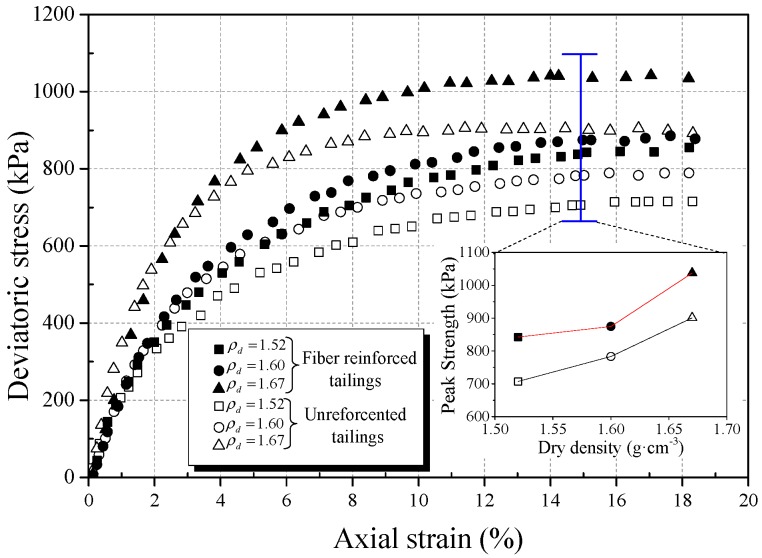
Effect of dry density on mechanical behavior of reinforced tailings silt (0.4 wt% fiber content).

**Figure 12:**
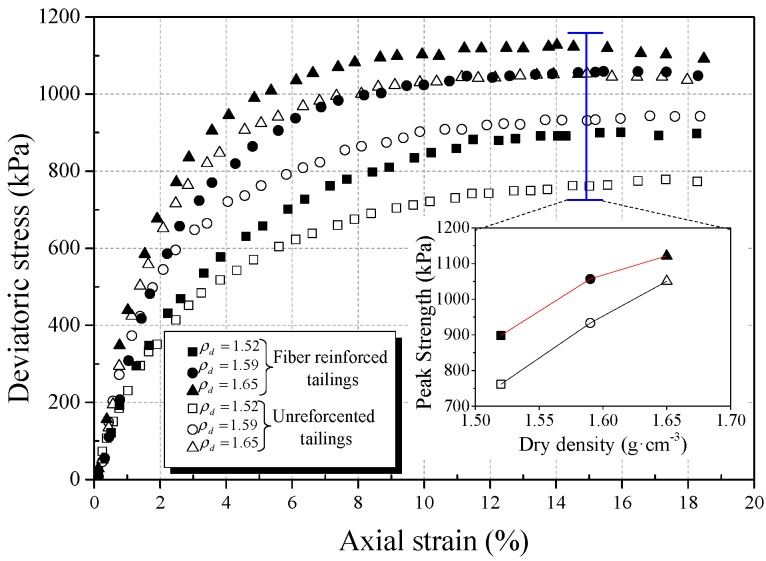
Effect of dry density on mechanical behavior of reinforced tailings sand (0.4 wt% fiber content).

In order to study the effect of the dry density of tailings on the strength behavior of BFRT, triaxial tests were carried out on three dry densities, different classes of reinforced and unreinforced tailings under the conditions of 6 mm fiber length, 0.4 wt% fiber content, and 400 kPa confining pressure. Figure 10, Figure 11 and Figure 12 show the stress strain curves and peak strength of the tailings under different dry densities.

It can be seen from Figure 10, Figure 11 and Figure 12 that the peak strength of tailings can be effectively improved with the increase in the dry density under the same conditions. This is normal because a higher dry density corresponds to a lower void ratio and a smaller pore size. This means that the interfacial effective contact area increases with increasing dry density, thereby increasing the interfacial bond strength to restrict the deformation. Dove et al. [42] and Tang et al. [43] obtained similar experimental results and stated that the effective contact area can directly affect the effect of reinforcement.

The experimental results show that the peak strength amplification of fiber-reinforced tailings clay, tailings silt, and tailings sand are 9.8%, 23.1%, and 24.8%, respectively, with the increase in dry density of this experiment.

Moreover, the amplification of the peak strength of reinforced tailings sand is obviously higher than that of tailings clay. The reasons are as follows:

1. The restraint effect of fibers on tailings mainly comes from the friction between particles and fibers.
2. With the increase in dry density, the contact area and biting force between tailings particles and fibers increase. It will increase the ability of the fibers to restrict the deformation of the tailings specimens.
3. The results of the microcosmic particle shape analysis show that the roughness and convexity of tailings sand particles are larger than tailings clay. The coarser the particles, the larger the friction between the particles and fiber filaments. Thus, the effect of reinforcement on BFRT is more significant for tailings sand.

### 4.3. Effect Confining Pressure on Strength Behavior of Tailings

**Figure 13:**
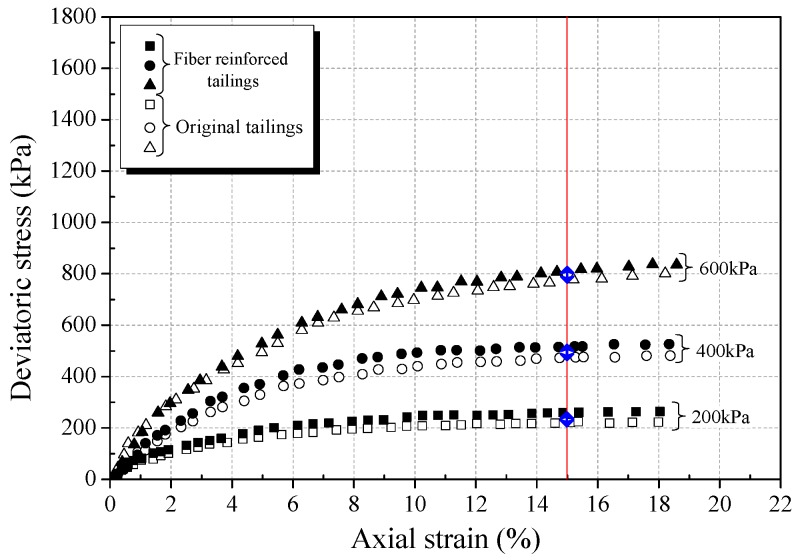
Stress strain curve for unreinforced and reinforced tailings clay (0.4 wt% fiber content).

**Figure 14:**
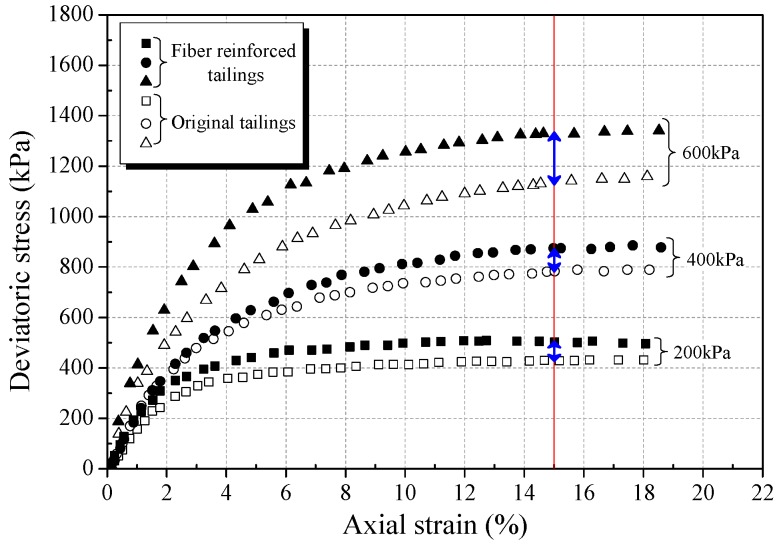
Stress strain curve for unreinforced and reinforced tailings silt (0.4 wt% fiber content).

**Figure 15:**
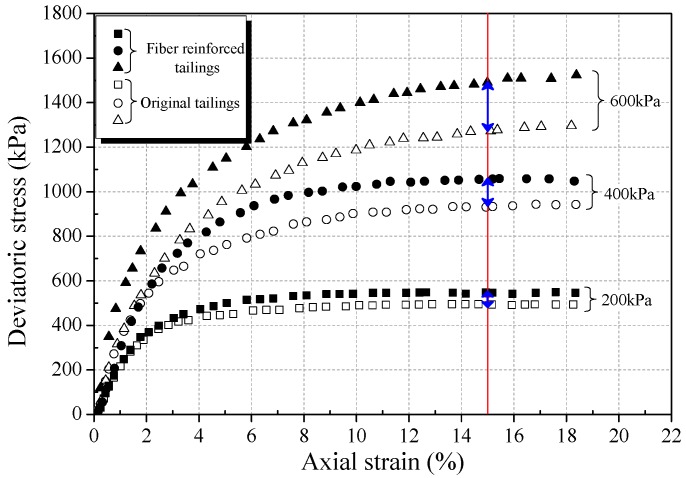
Stress strain curve for unreinforced and reinforced tailings sand (0.4 wt% fiber content).

Increasing the confining pressure improves the strain hardening and failure toughness of fiber-reinforced soil [10,39]. In order to study the effect of the confining pressure on the mechanical properties of fiber tailings, triaxial tests under three confining pressures (200 kPa, 400 kPa, 600 kPa) were carried out on tailings clay, tailings silt, and tailings sand with dry densities of 1.52 g·cm^−3^, 1.60 g·cm^−3^, and 1.59 g·cm^−3^ respectively, under the condition of 0.4 wt% fiber content and 6 mm fiber length. Figure 13, Figure 14 and Figure 15 show the stress-strain curves of three classes of tailings under different confining pressures.

From Figure 13, Figure 14 and Figure 15, it can be seen that the strain hardening degree and failure toughness of fiber-reinforced tailings can be improved with the increase in confining pressure, consistent with findings of the Consoli et al. [13] and Shao et al. [44]. With the increase in confining pressure from 200 to 600 kPa, the peak strength of BFRT increases significantly, and the three classes of tailings all transit to the strain hardening type. This is because the tailings are relatively loose under low confining pressure, and there is a large number of pores in tailings. With the increase in confining pressure, the size of the pores in the tailings decreases, and the biting force between fiber filament and tailings particles increases, enhancing the effect of fiber reinforcement.

**Figure 16:**
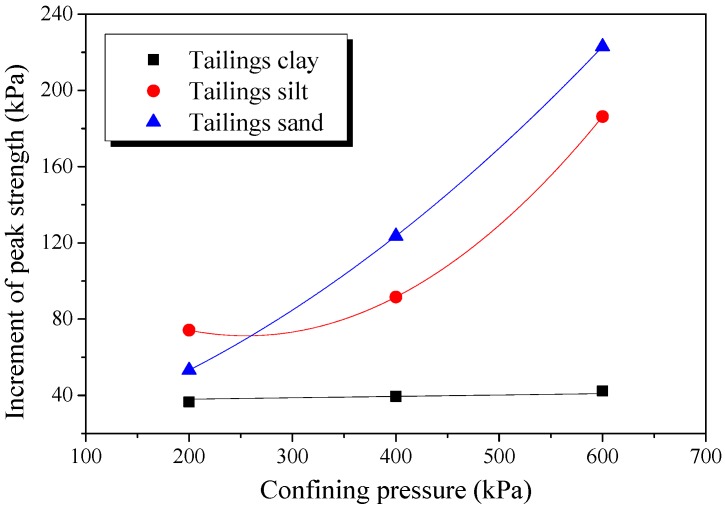
Increment of the peak strength of reinforced tailings under different confining pressure.

The increment of peak strength (interval of blue arrows in Figure 13, Figure 14 and Figure 15) with confining pressure for three classes of BFRT is shown in Figure 16. Under 200 kPa, 400 kPa and 600 kPa confining pressures, the increments of the peak strength of reinforced tailings clay are 36.6 kPa, 39.5 kPa, and 42.3 kPa, tailings silt is 74.2 kPa, 91.6 kPa, and 186.3 kPa, and the tailings sand is 53.2 kPa, 123.6 kPa, and 223.0 kPa compared to unreinforced tailings. The increment shows an increasing trend with the increase in confining pressure. The peak strength of reinforced tailings sand and tailings silt are greatly affected by confining pressure, while the reinforced tailings clay is less affected. The reasons for this are as follows: the roughness of particles increases with the increase in the particle size obtained from the particle shape analysis, the interfacial biting force between particles and fibers increases under confining pressure, and the ability of fibers to restrict the deformation of soil particles becomes stronger. Therefore, the influence of confining pressure on coarse tailings is higher than that on fine tailings.

## 5. Interface Characteristics of Fiber-Reinforced Tailings

**Figure 17:**
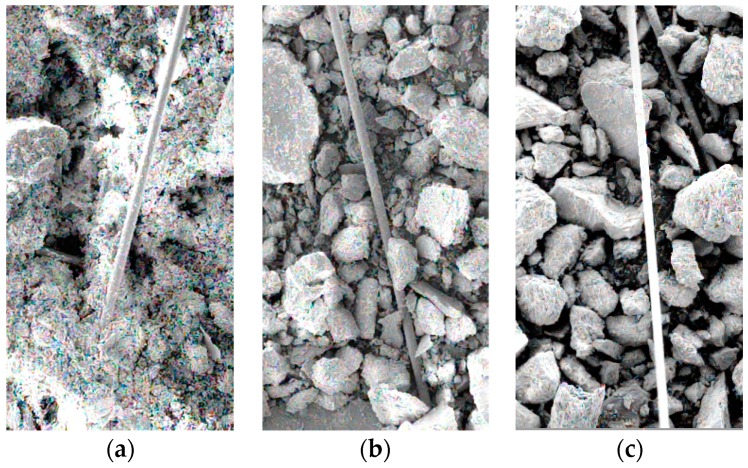
SEM images of fiber monofilaments in BFRT specimens. (**a**) Fiber-reinforced tailings clay, (**b**) fiber-reinforced tailings silt, (**c**) fiber-reinforced tailings sand.

SEM images of the morphology of the basalt fiber monofilaments in the BFRT specimens are presented in Figure 17. From Figure 17a, it can be seen that the fiber is wrapped by tailings clay which produces adhesive force between fiber and tailings particle. Figure 17b,c show that the fiber surface is bitten by coarse tailings particles with obvious edges and corners which contributes to biting force. The biting force makes the fibers difficult to slide and can bear tensile stress compared to adhesive force.

**Figure 18:**
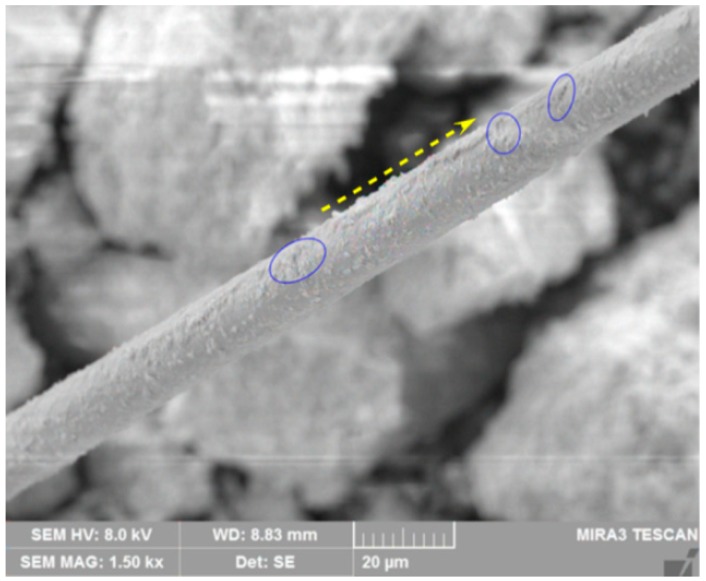
Grooves and scratches formed on the fiber surface.

Figure 18 presents the surface of the fiber monofilaments in the shear failure specimen by SEM. It can be seen from the figure that the surface of the fiber is roughness, and there are obvious grooves and scratches as the marked area of Figure 18. The reasons may be as follows: (1) As the fibers were mixed or mixture samples were compacted during preparation, the angular tailings particles impacted and abraded the fiber surface, causing the scratches. (2) During the shear test, the fibers restricted the deformation of the specimens causing the relative slipping between the fibers and the particles. When the slip occurs between the angular particles and the fibers, it will cause the surface of the fibers to peel off and form grooves under the action of friction and extrusion force. All these grooves and scratches on the fiber surface will lead to an increase in roughness and the friction coefficient. The existing research indicates that the fiber sliding resistance is strongly dependent on the fiber surface roughness [45]. Therefore, the existence of these grooves and scratches can improve the effect of fiber reinforcement.

**Figure 19:**
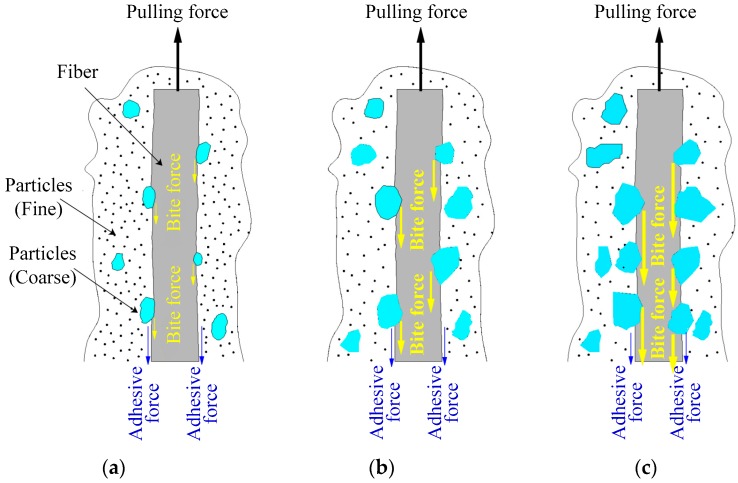
Sketch of interfacial mechanics behaviors between tailings particles and fibers. (**a**) Tailings clay, (**b**) tailings silt, (**c**) tailings sand.

According to the experimental analysis, the interfacial mechanics behaviors of different classes of tailings particles and fibers are shown in Figure 19. The interaction between fibers and particles is mainly caused by two forces: Adhesive force (cohesive force and friction force) and biting force. Although both of these forces exist in the BFRT, as the tailings clay is mainly composed of fine particles the adhesive force is the main force between fibers and tailings particles. In contrast, the tailings sand contains a large number of coarse particles with distinct edges and corners which will produce biting forces to restrict the deformation when the specimens are under load. Based on the analysis of mechanical tests results, we can conclude that the biting forces play a dominant role in the BFRT. This can also illustrate the reason why the reinforcement effect of tailings sand and tailings clay is better than that of tailings clay.

The interfacial behavior of fiber monofilaments can be extended to the fiber network structures. These randomly distributed discrete fibers act as spatial network structures to interlock the particles and help to restrict the displacement under the action of adhesive force and biting force.

## 6. Conclusions

In this investigation, basalt fibers were deliberately selected as the material for tailings reinforcement. The effect of fiber length, fiber content, particle size, dry density, and confining pressure on the mechanical behaviors of the BFRT were analyzed. The interface characteristics between fibers and particles were additionally investigated by SEM. The following conclusions were obtained:

1. Basalt fibers can be used as reinforcing materials for tailings disposal. Fiber reinforcement can effectively improve the mechanical strength of the tailings. With the increase in fiber content and fiber length, it tends to form more network structures in test specimens. The network structures can effectively bear the pulling force and prevent the destruction of the tailings specimens. The peak strength of reinforced tailings improves with the increase in fiber length and content.
2. With the increase in particle size the convexity and roughness increases and the sphericity decrease of the shape features. Large particles with an angular surface generally have a better effect for fiber reinforcement. Therefore, the effect of fiber reinforcement for test tailings is: tailings sand > tailings silt > tailings clay.
3. Increasing the dry density can effectively improve the mechanical properties of tailings. The shear strength of reinforced fine sand tailings is obviously higher than that of silt clay, due to the influence of dry density. Moreover, the amplification of the peak strength of reinforced tailings sand and tailings silt is obviously higher than that of tailings clay.
4. The increment of the strength shows an increasing trend with the increase in confining pressure. Due to the influence of the particle shape, fiber-reinforced tailings sand and tailings silt are more sensitive to confining pressure compared to tailings clay.
5. The interfacial interaction between fibers and tailings particles is mainly affected by particle shape. The interaction is mainly caused by adhesive force and biting force. The biting force occurs at the interface between angular particles and fibers surface seem to be the dominant mechanisms controlling the reinforcement benefit.

These conclusions are significant both for the understanding of the basic mechanical behaviors of BFRT, and for application in tailings dam projects. The actual reinforcement effect and economic problems related to using BFRT in engineering practice need to be further studied.

## Figures and Tables

**Table 1:** Technical specifications of the basalt fibers [37].

Fiber Type	Density/g·cm^−3^	Fracture Strength/MPa	Tensile Strength/MPa	Elastic Modulus/GPa	Acid and Alkali Resistance
Monofilament	2.7	3200	2650	89	Excellent

**Table 2:** Physical index of the test tailings.

Sample No.	Tailings Class	Specific Gravity	Plastic Limit (%)	Liquid Limit (%)	Plastic Index	C_u_	C_c_
#1	tailings clay	2.98	4.72	23.67	18.94	6.01	1.02
#2	tailings silt	3.03	10.01	18.81	8.8	8.94	4.1
#3	tailings sand	3.02	/	/	/	3.15	1.03

^1^ tailings clay are clay sized particles, tailings silt are silt sized particles, tailing sand are sand sized particles.

**Table 3:** Main chemical composition of the test tailings.

Composition	Sn	Cu	Pb	Zn	Bi	Sb	Fe	S	SiO_2_	CaO	Al_2_O_3_	MgO
Content (%)	0.35	0.04	0.03	0.06	0.05	0.03	10.04	0.36	40.16	19.44	6.93	2.85

**Table 4:** Test schemes.

Test Group	Fiber Length (mm)	Fiber Content (wt%)	Dry Density (g·cm^−3^)	Confining Pressure (kPa)
Group 1	3, 6, 9	0.2, 0.4, 0.6	1.52(#1), 1.60(#2), 1.59(#3)	400
Group 2	6	0.4	1.40, 1.52, 1.61 (#1), 1.52, 1.60, 1.67 (#2), 1.52, 1.59, 1.65 (#3)	400
Group 3	6	0.4	1.52(#1), 1.60(#2), 1.59(#3)	200, 400, 600
